# Supplementary material for: Splice-Junction-Based Mapping of Alternative Isoforms in the Human Proteome
Source: Cell Rep. Author manuscript; Available in PMC 2020 Jan 15. (PMC6961840; doi:10.1016/j.celrep.2019.11.026)
Supplement: 3 [file NIHMS1546469-supplement-3.zip › DF2/PXD006675/RightVentricle-375-Q9UKM9-AASAIYR.pdf]

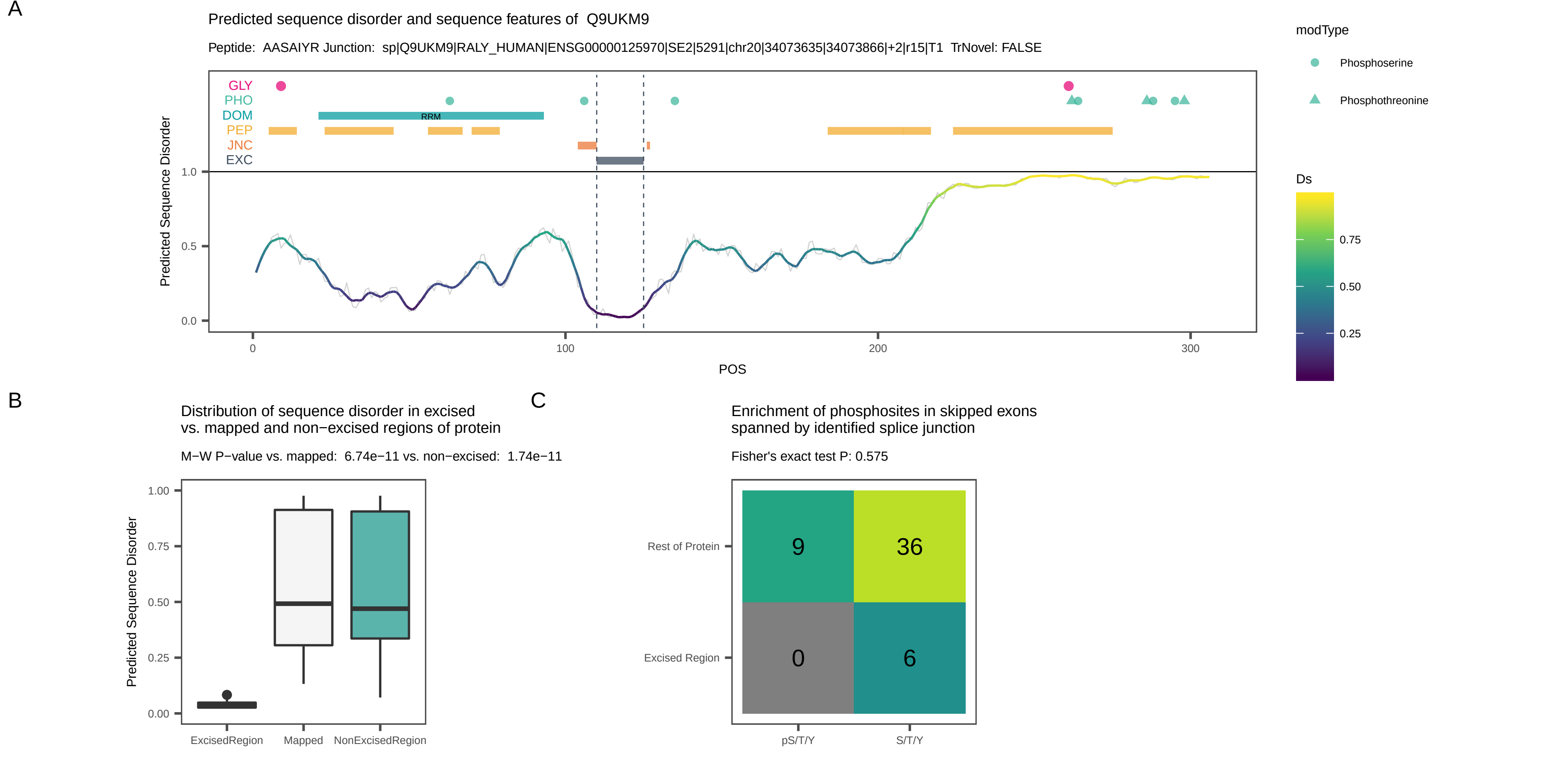
A
Predicted sequence disorder and sequence features of Q9UKM9
Peptide: AASAIYR Junction: sp|Q9UKM9|RALY_HUMAN|ENSG00000125970|SE2|5291|chr20|34073635|34073866|+2|r15|T1 TrNovel: FALSE
GLY
PHO
DOM
PEP
JNC
EXC
RRM
Predicted Sequence Disorder
1.0
0.5
0.0
0
100
200
300
POS
modType
Phosphoserine
Phosphothreonine
Ds
0.75
0.50
0.25
B
Distribution of sequence disorder in excised vs. mapped and non−excised regions of protein
M−W P−value vs. mapped: 6.74e−11 vs. non−excised: 1.74e−11
Predicted Sequence Disorder
1.00
0.75
0.50
0.25
0.00
ExcisedRegion
Mapped
NonExcisedRegion
C
Enrichment of phosphosites in skipped exons spanned by identified splice junction
Fisher's exact test P: 0.575
Rest of Protein
Excised Region
9
36
0
6
pS/T/Y
S/T/Y
